# Synopsis of the Clinic Lectures of Prof. John W. Jones, in the Atlanta Medical College

**Published:** 1860-09

**Authors:** 


### ARTICLE II.

*Synopsis of the Clinic Lectures of* Prof. John W. Jones, in the Atlanta Medical College. Session 1860.

### *Dentition and Diarrhœa.*

May 28.—Negro child, male. This child is some fifteen months old—has had ordinary diarrhœa occasionally for several weeks past—find its pulse moderately full and frequent—head hot—extremities cool—mouth and tongue dry, and, as you perceive, it appears feeble, but not much emaciated, but will probably soon become so. It may prove a tedius case, and even terminate fatally. Such cases require, and ought to receive prompt and skillful attention. A number of teeth have appeared, and others are about to protrude. It has, we learn, for some time past, been indulged in various dietetic improprieties; its mother is not present, and we are, therefore, not informed whether or not it is suffering from premature ablactation, a practice as culpable as it is common, but less so, perhaps, with blacks than whites, as a general rule; advised to condemn it. The current of nature usually runs in the right direction. Of the nature of the alvine discharges in this case, can learn nothing, except that the child has a *bowel complaint*, and cannot, therefore, prescribe judicially; advised milk or milk and bread diet, in proper quantities, and at proper intervals, alternated with well boiled rice, or milk and rice; that his gums be freely incised over the teeth about to appear, and hydrarg cum creta in alternate doses, say two grains, every other night for one week, or until a favorable change is observed, and to be resumed if the present symptoms reappear. Vesication behind and beneath the ears would likely be of service in this case. Anodynes and narcotics, and especially opium, as well as astringents, should, if at all, be sparingly employed. Ordinarily, it is better to avoid both, lest by their use we promote that well known tendency to metastatic meningetes and hydrocephalus as a sequence.

### *Dysmenorrhœa and Leucorrhœa.*

May 30.—Negro woman, the property of Col. ———, of this city, aged about thirty-eight years—of apparently robust constitution, and with no external manifestations of disease, except considerable tumefaction (without pain) of the right superior extremity, from hand to shoulder. Pressure on the spinal column between the scapulæ produces as you observe, pain and flinching. She is the mother of a child, now some seven or eight years old; has menstruated regularly, but not healthily, for several years past; just preceding and during her monthly periods, suffers from nausea, sometimes vomiting; pains in the loins and uterus, and discharges dark clots, &c. During the catamenial intervals she is much troubled with leucorrhœa; suspects the existence of vaginities, possibly sub acute inflammation of the os tincœ.

Advises scarifications and cups repeatedly over the tender portion of the spine; the same over the lumbar region; then vesication over those localities; and five grains of blue mass night and morning, every third or fourth day, for one month; anticipates the necessity of additional treatment; recommends an emulsion of copaiva, tincture of iodine and solution of nitrate of silver, separately, as injections for vagenites, ordinary leucorrhœa.

### *Partial Insanity.*

June 1st.—Negro woman, age 20 years, was carried last winter from the mountain region of Virginia to South Alabama, changed owners, and was never a field-laborer until the past spring. In May last had symptoms of malarial fever, and took quinine freely from the hands of the plantation overseer: severe headache and mental aberrations ensued; and she now labors under constant apprehensions (without cause) of severe chastisement of her present master, who is humane and indulgent to his slaves; has spells of crying, lamentations, &c., for which she can assign no plausible reason, and, as you perceive, her countenance is indicative of profound melancholy, and she appears to be considerably debilitated. Her's is not an idiotic appearance; it is rather that of partial mania; circulation ordinary; do not think that these symptoms are feigned, and for these hallucinations or perturbations, advise a quiet room, cooling applications to the scalp, scarifications and cups to the temples, mild laxatives, light diet, kind treatment, and friendly assurances.

June 10.—In the above case a gradual improvement was perceptible in a few days, and the woman is now well in body and mind. We are inclined to impute her condition to the combined influences of nostalgia and over doses of quinine.

### *Aphrodesiasmus.*

A negro man, age 23, of medium size, and more than ordinary intelligence in boyhood, and now of temperate habits, except an almost incontrolable venereal appetite, which developed itself as early as eight years of age, according to his report, and which has been indulged *ad libitum*, and to great excess, up to a very recent date. He is now in a most pitiable condition. This man is now in a state of extreme nervous and muscular prostration, feeble circulation, flabby muscles, essential loss of memory; indeed in a state of mental as well as physical impotency and imbecility, his sight dim and confused, his countenance dull and irresolute, indigestion with constipated bowels and supposed enlargement of the prostate gland.

Treatment—Abstinence for a season in regard to the indulgence of his ruling passion, with tonics and *good living;* then temperance and anaphrodesiacs and if needs be orcheotomy.

Does this state of things arise from excessive sensibility of the genital organs, or from mental and moral depravity, or in part from all,conjointly? we think the latter.

### *Convulsions and Mental Derangement.*

You observe before you a robust muscular man between thirty-five and forty years of age, and exhibiting a physiognomy and countenance more idiotic than maniacal, and his actions also tend to the confirmation of this opinion; nevertheless, fifteen years since he was an intelligent and valuable blacksmith. In a rencounter with a fellow smith he received a severe blow from a hammer-shaped piece of iron upon his left parietal protuberance, which instantly felled him to the floor, and deprived him of conciousness and voluntary motion; both tables of the skull, at the point mentioned, were driven in upon the brain; gradually he recovered *partially* from the injury, but has been the victim of frequent convulsions from a few weeks after the blow was received to the present time, and he is now in a condition of almost entire mental imbecility. In this case fracture of the skull, with depression of bone, was produced. The trephine was not used nor was the depressed portions of bone elevated—unpardonable neglect.

The class was referred to a report of a number of similar cases by Prof. A. W. Dudley, as given to the public through the medium of the Transylvania Medical Journal, several of which patients were permanently restored mentally and physically by the use of the trephine in the hands of that distinguished surgeon.

In many of the cases operated upon by Prof. Dudley, spiculæ of bone were found projecting from the depressed portions of the skull into the brain, and surrounded by pus or disorganized cerebral matter. The case presented exhibits the cicatrix of the original injury, and is the index for the centre of the trephine, and the use of that instrument is recommended.

*Asthma.*

A gentleman, age 29 years, has suffered severely—with short intervals between the fits—from this affection for fifteen years. Many remedies have been used with but little if any mitigation of the symptoms, and that only temporarily. Do not regard asthma so much a disease as the symptom of a disease, the primary lesion of which may exist in the lungs, or more remote—think that remedies are too commonly addressed exclusively to the lungs, and hence their frequent, or almost uniform failure to relieve the patient—that the examination of asthmatics is usually too superficial—that such investigations fall short of the mark—do not reach the punctum saliens, and hence the errors in diagnosis, and the blunders in therapeutics—that a knowledge in all the existing facts in each particular case of metastasis and of reflex nervous action can solve the true pathology of the disease, and point to the indications of scientific and successful treatment.

Among the local lesions that give rise to asthmatic symptoms, may be enumerated paralysis of the pneumogastric nerves, and that is but a sequence of a more remote link of the etilogical chain. Emphysema, pneumothorax, spasmus glottidis, various cardiac affections, bronchitis, flatulency, extreme fullness of the stomach—anything calculated to crowd or congest the lungs, or to irritate the bronchial lining, may induce a fit of the disease.

